# Changes of Functional, Morphological, and Inflammatory Reactions in Spontaneous Peripheral Nerve Reinnervation after Thermal Injury

**DOI:** 10.1155/2022/9927602

**Published:** 2022-02-01

**Authors:** Xing Yu, Chang Liu, Caihong Ji, Cameron Lenahan, Yuanjian Fang, Yong Wang, Anwen Shao

**Affiliations:** ^1^Department of Surgery, The Second Affiliated Hospital, Zhejiang University School of Medicine, Hangzhou, China; ^2^College of Medicine, Zhejiang University, Hangzhou, China; ^3^Department of Neurology, The First Affiliated Hospital, College of Medicine, Zhejiang University, Hangzhou, China; ^4^Center for Neuroscience Research, Loma Linda University School of Medicine, Loma Linda, CA, USA; ^5^Burrell College of Osteopathic Medicine, Las Cruces, NM, USA; ^6^Department of Neurosurgery, The Second Affiliated Hospital, Zhejiang University School of Medicine, Hangzhou, China

## Abstract

In recent decades, the use of energy-based devices has substantially increased the incidence of iatrogenic thermal injury to nerves (cauterization, etc.). While recovery of the nerve after thermal injury is important, the changes in neural structure, function, and peripheral inflammatory reactions postinjury remain unclear. This study is aimed at demonstrating the changes mentioned above during the acute, subacute, and chronic stages of nerve reinnervation after thermal injury. Spontaneous reinnervation was evaluated, including the neural structures, nerve conduction abilities, and muscle regeneration. These effects vary depending on the severity of thermal injury (slight, moderate, and severe). Peripheral inflammatory reactions, as impediments to reinnervation, were found in significant numbers 3 days after thermal injury, exhibiting high expression of IL-1*β* and TNF-*α*, but low expression of IL-10. Our findings reveal the pathogenesis of peripheral nerve reinnervation after thermal injury, which will assist in selecting appropriate treatments in further research.

## 1. Introduction

Energy-based devices (EBDs) have been widely adopted for hemostasis in recent decades due to the easy application and effective reduction in blood loss [1, 2]. However, the use of EBDs is also controversial due to the high temperature of the head and the lateral thermal spread during activation. The incidence of iatrogenic thermal injury has reportedly increased, and the heat has gradually become a common cause of peripheral nerve injury [3].

Nerve thermal injury, as a less reversible type, was associated with obvious histologic damage [4–6]. It has been revealed that the peripheral nerves manifest as a reversible conduction block when exposed to lower-grade thermal injury but manifest with axonal degeneration at higher temperatures [7]. What is more, there is a decrease in the manifestation of electromyographic (EMG) changes in amplitude with a concomitant increase in latency during acute thermal nerve damage [8]. However, it is unclear whether peripheral nerves could develop spontaneous reinnervation after thermal injury. It is also unclear how the functional recovery differs when considering the severity or the time of recovery after thermal injury.

It has been demonstrated that nerve reinnervation is evaluated by assessing not only the reconstruction of the neural and muscular complex but also the improvement of conductive function [9]. EMG and the number of neuromuscular junctions (NMJ) in the skeletal muscles are critical indices in evaluating the recovery of nerve conduction abilities [10, 11]. Furthermore, the role of peripheral inflammation and cytokine release was found and is reportedly active during nerve regeneration [12, 13].

This study aimed at elucidating the nerve's morphological and functional changes, as well as the peripheral inflammatory reactions that occur during spontaneous reinnervation following thermal injury in a rat model. We hypothesize that the consequence of reinnervation differs from the severity of thermal injury, presents in a time-dependent manner, and may be regulated by peripheral inflammatory reactions.

## 2. Methods

### 2.1. Surgical Procedures

The study involved 48 Sprague-Dawley (SD) male rats weighing 200-220 g. As described in the previous study, all rats were anesthetized with intraperitoneal injection of pentobarbital (40–80 mg/kg, 10 times dilution by physiological saline) and maintained on spontaneous ventilation [14]. As described in the previous study, each sciatic nerve was exposed via separation of the biceps femoris and the tensor fascia latae [9]. The right sciatic nerve was exposed to slight, moderate, and severe thermal injury, while a sham operation was performed on the right limb without any thermal injury as the control. The harmonic scalpel (HS; Ethicon Endo-Surgery, Inc.), as one of the EBDs, was activated for 10 seconds at power setting of 5. The head of the HS touched the nerve directly as the severe injury group, whereas the distances between the nerve and HS were controlled 1 and 3 mm in the moderate and slight injury groups, respectively. An infrared camera was used to improve the validity of the experimental model, quantifying the temperature based on infrared images so as to classify the thermal injury delivery as slight, moderate, and severe. Two surgeons performed the operation. While one of them used millimeter paper to verify that the distances between nerve and HS were consistent with the expectation, another surgeon manipulated the dissection part.

### 2.2. Schematic Diagram of Experimental Design

**Figure 1:**
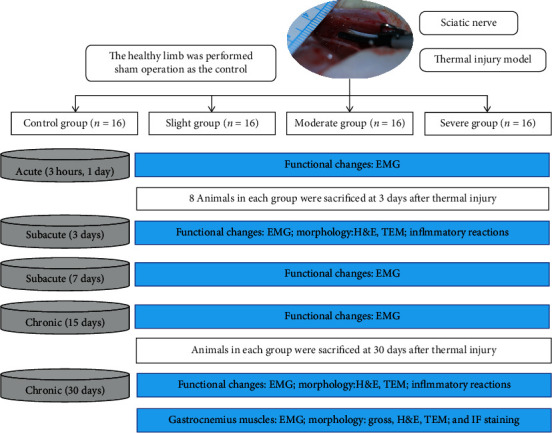
Schematic diagram of experimental design and animal group classification. EMG: electromyographic analysis; TEM: transmission electron microscopy; H&E: hematoxylin and eosin staining.

The functional, morphological, and peripheral inflammatory changes were recorded at the acute (3 hours and 1 day), subacute (3 and 7 days), and chronic (15 and 30 days) stages after thermal injury. Functional changes of the nerve were evaluated via EMG, including signal amplitude, latency, and nerve conduction velocity (NCV). The transversely morphological changes, including the structures of the sciatic nerve and gastrocnemius muscle, were recorded by using a light scope of hematoxylin and eosin (H&E) staining, immunofluorescent (IF) staining, and transmission electron microscopy (TEM). The changes in serum cytokine levels (TNF-*α*, IL-1*β*, and IL-10) were analyzed via the enzyme-linked immunosorbent assay (ELISA) to assess the peripheral inflammatory reactions of the nerve. Animals were sacrificed via intracardiac injection of potassium chloride solution at 3 days (representing subacute evidence) and 30 days (chronic evidence) after thermal injury (Figure 1).

### 2.3. Experimental Group Division

**Figure 2:**
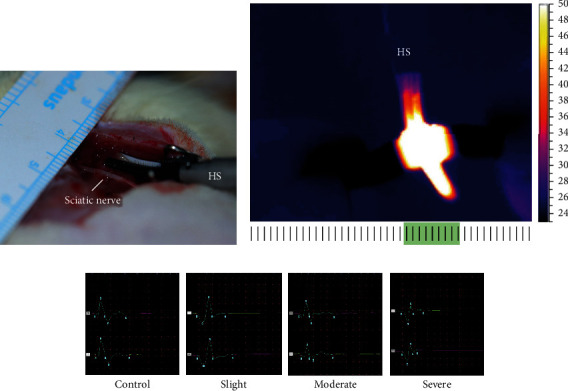
Establish the different degrees of severity of peripheral nerve thermal injury. (a) HS is activated parallel to the sciatic nerve to modify acute thermal injury. (b) Tissue heat map during HS activation based on infrared images with an infrared camera. (c) Features of EMG changes after thermal injury show that the amplitude decreased (order: severe > moderate > slight ≥ control) and the latency was prolonged (severe > moderate > slight ≥ control). Green band in (b) indicates the lateral thermal spread. HS: harmonic scalpel.

The rats were divided randomly into four groups according to the severity of the thermal injury. The severity was dependent on the parallel distance from the nerves to HS. For instance, in the severe injury group, the head of the HS touched the nerve directly (the distance is 0 mm), whereas the distances between the nerve and HS were 1 and 3 mm in the moderate and slight injury groups, respectively. Sham operation was designed as nerve exposure, but without thermal injury as the control group. The results of the EMG changes (detected immediately and retested at 3 hours after thermal injury) were recorded to verify the severity of nerve thermal injury (the control, slight, moderate, and severe groups) (Figure 2). These procedures were reviewed and approved by the Institutional Review Board (Ethics Committee, the Second Affiliated Hospital of Zhejiang University, School of Medicine) (approval number 2019-393).

### 2.4. EMG Analysis

As described previously in the protocol, the sciatic nerves were exposed in the anesthetized animal for electrophysiological testing [15]. The stimulating cathode, composed of a stainless-steel monopolar needle, was placed at the sciatic nerve trunk, and the parallel distance was 10 mm between the two cathodes. The motor response was recorded continuously and distally using a unipolar steel needle electrode inserted into the gastrocnemius muscle. Additionally, NCV, amplitude, and latency were recorded using a digital neurophysiological system (Neuro-MEP-Micro, Neurosoft Ltd., 5, Voronin Str., Ivanovo, 153032, Russia). The time for the electrical impulse to travel from the stimulation to the recording site was measured as the latency. Amplitude was calculated as the areas from baseline to the maximal negative peak. NCV was obtained by dividing the distance of 10 mm by the difference in the latency time.

### 2.5. Transmission Electron Microscopy (TEM)

TEM was conducted to record structural changes in the nerve, particularly the morphological characteristics of myelinated axons and myelinated sheaths. A portion of the nerve was removed, fixed in 2.5% glutaraldehyde overnight in the refrigerator, and then washed in phosphate-buffered saline (pH 7.2) for 3 times at 15 min each. Then, it was placed in 1% osmium tetroxide and washed 3 times for 1 h each. After, the samples were stained with 4% uranyl acetate for 30 min, dehydrated by a series concentration of ethanol, and then embedded in resin mixture (70, 80, 90, and 95% ethanol, 10 min each). This was sectioned and observed under a TECNAI-10 transmission electron microscope (Phillips) [16].

### 2.6. Immunofluorescent (IF) Staining

Tissue samples were sectioned (4 *μ*m thickness, CM1850, LEICA, Germany), stained using IF markers, and observed under a fluorescence microscope (ECLIPSE 80i, Nikon, Japan). Nuclei were stained blue using 4,6-diamidino-2-phenylindole (DAPI, Biosynthesis Biotechnology, China). Primary antibodies against the neurofilament (GT114, mouse anti-rat, GeneTex, Texas, USA) were used to identify axons and NMJ in the nerves and gastrocnemius muscles, respectively. Goat anti-mouse (red, Thermo Fisher Scientific, USA) was used as the secondary antibody.

### 2.7. Enzyme-Linked Immunosorbent Assay (ELISA)

Productions of TNF-*α*, IL-1*β*, and IL-10 were quantified using ELISA kits (Yili Biology, Shanghai, China) following the manufacturer's instructions. Briefly, a total of 5 mL of blood was collected from the exposed heart of the deeply anesthetized animals and incubated at room temperature for 30 min. The serum was obtained after centrifuging the whole blood at 2000 rpm at 4°C for 15 min and then analyzed using an ELISA kit. The concentrations of TNF-*α*, IL-1*β*, and IL-10 were measured through correlation with a standard curve. Blank disks were used as the control.

### 2.8. Statistical Analysis

Data were expressed as mean ± standard error, and statistical analysis was performed by Student's *t*-test, Kruskal-Wallis test, Fisher's precise test, and one-way analysis of variance (ANOVA) using the SPSS software (IBM SPSS Statistics, USA). Data were illustrated using the GraphPad Prism (GraphPad Software Inc., San Diego, CA, USA).

## 3. Results

### 3.1. Nerve Electrophysiological Changes after Thermal Injury

**Figure 3:**
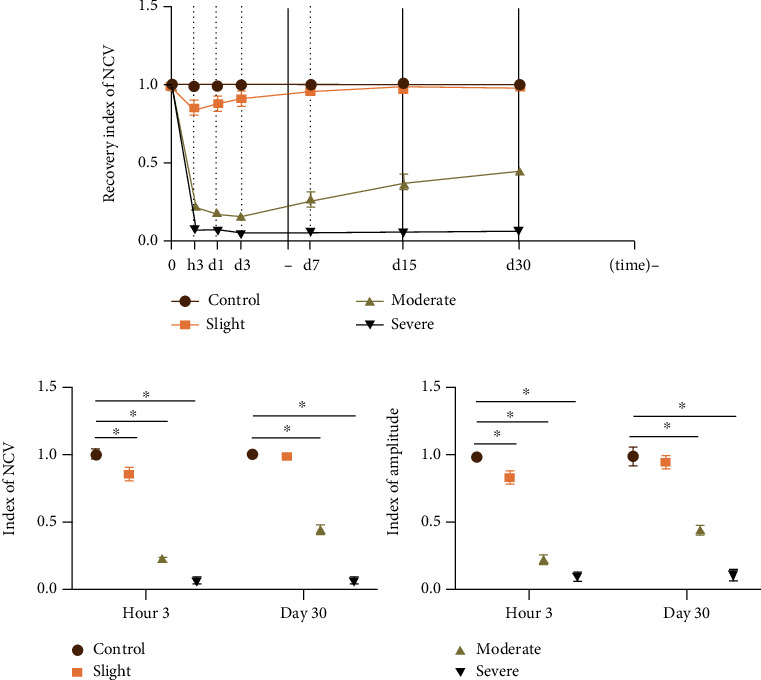
Nerve electrophysiological changes according to EMG analysis after slight, moderate, and severe thermal injury. (a) Recording the recovery of the NCV index in each time point (acute, subacute, and chronic) from 3 hours to 30 days after thermal injury. (b) Comparison of EMG changes in the three groups; indices of NCV and amplitude were decreased significantly after thermal injury, and the severity of injury was in the order of severe > moderate > slight ≥ control. NCV: nerve conduction velocity. ∗ represents *P* < 0.05.

Nerve electrophysiological changes, such as NCV, amplitude, and latency, were recorded via EMG to evaluate the nerve conductive abilities. The nerve conductive abilities were destroyed, which was exhibited as the decrease in NCV and amplitude, as well as latency prolongation after thermal injury. The NCV and amplitude indices vary from 0 to 1, with 0 and 1 corresponding to complete dysfunction and normal function, respectively. The NCV index was measured in each time point (acute, subacute, and chronic) after thermal injury to assess spontaneous nerve reinnervation recovery. In the slight group, the NCV index was impaired immediately but began to recover 3 hours after thermal injury. However, the NCV index decreased immediately after thermal injury in the moderate group and continued to decline over the following 3 days. It should be noted that the NCV index began to increase from the 3^rd^ to 30^th^ day after thermal injury. In the severe group, the NCV index was also significantly decreased but could not recover until 30 days after thermal injury. As depicted in Figure 3, the NCV index was significantly decreased at 3 hours after thermal injury, exhibited as 0.85 ± 0.04, 0.23 ± 0.04, and 0.06 ± 0.07 in the slight, moderate, and severe groups, respectively. The NCV index was recorded at 30 days after thermal injury to evaluate the recovery of spontaneous nerve reinnervation, presenting as 0.97 ± 0.05, 0.45 ± 0.04, and 0.06 ± 0.08 in slight, moderate, and severe groups, respectively, with significant difference. There was no significant difference in the comparison of EMG changes within groups between 3 hours and 30 days after thermal injury.

### 3.2. Nerve Pathological Changes after Thermal Injury

**Figure 4:**
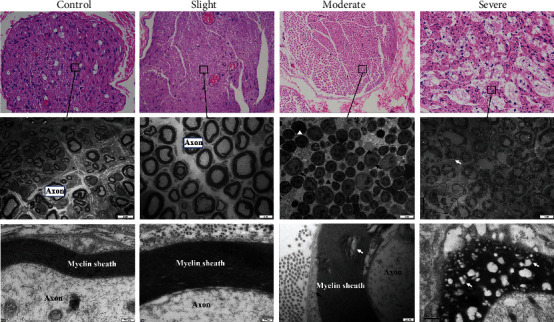
Nerve pathological changes assessed via light and electron microscopy after slight, moderate, and severe thermal injury. (a) Getting histologic results via light microscopy, the black box in the severe group indicates characteristics of coagulation and homogenization (H&E staining, 200x). (b, c) Comparison of the pathological changes of axons and myelin sheaths (TEM). White triangles indicate the hyperpigmentation of axonal fibers. Black arrows indicate the loose myelin sheaths with reduced anchoring particles. Black triangle indicates macrophages adjacent to the myelin sheath. White arrows indicate bubbles in myelin sheaths. TEM: transmission electron microscopy.

Nerve pathological changes were recorded via light and electron microscopy at 3 days after slight, moderate, and severe thermal injury (Figure 4). In subjective evaluation, there was no difference in neural appearance between the slight thermal injury group and the control group. Under light microscopy, the axons were frequently concentrically wrapped by the Schwann cells. TEM showed myelinated axons and a thick myelin sheath, in which numerous anchoring particles were observed maintaining the integrity of the myelin sheath. However, in the moderate thermal injury group, the neural appearance showed that myelin sheaths were loose and accompanied by lessened anchoring particles. The membrane of the myelin sheaths demonstrated effusion, which was considered a result of inflammatory reactions. The number of macrophages was increased, and they showed a tendency to be near the myelin sheath. In the severe thermal injury group, neural features of coagulation and homogenization, accompanied by peripheral inflammatory reactions, were observed by light microscopy. The TEM revealed that the number of intact axon fibers was significantly decreased in the same magnification. Multiple bubbles were detected in the myelin sheaths, and these bubbles destroyed the compact structure of the myelin sheath. The number of anchoring particles was significantly decreased in the same magnification, and the myelin sheath became loose and irregular. It can be speculated from the exhibitions above that these damaged structures impair the transport of neural signals (Table 1). The exhibitions of nerve pathological changes at 30 days were consistent with the results at 3 days after slight, moderate, and severe thermal injury.

### 3.3. Morphological Changes of the Gastrocnemius Muscle at 30 Days after Thermal Injury

**Figure 5:**
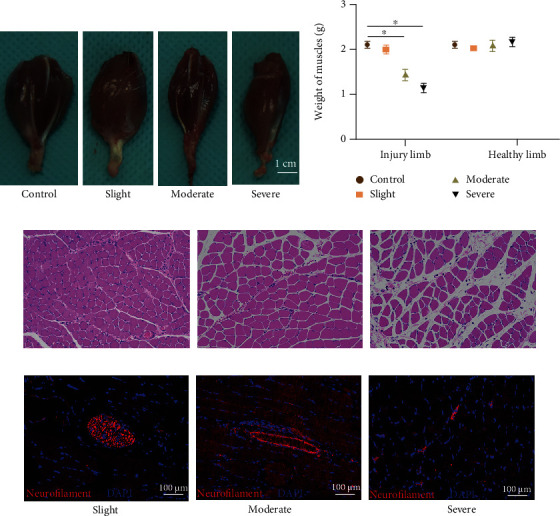
Macro- and micromorphological changes of gastrocnemius muscles at 30 days after thermal injury. (a) Gross observation of gastrocnemius muscles. (b) Comparison of the weight changes in the four groups; the order of weights of the injured limb are listed as follows: severe group < moderate group < slight group ≤ control group with significant difference. (c) Recording the morphology of muscle cells by microscopic observation (H&E staining, 100x). (d) Getting the number of NMJ in gastrocnemius muscles by IF staining (indexes: neurofilament and DAPI). Nuclei were stained blue using DAPI, and neural fibers stained red using antineurofilament. ∗ represents *P* < 0.05. NMJ: neuromuscular junctions; IF: immunofluorescent.

The macro- and micromorphological changes of the gastrocnemius muscle are recognized as a vital index to evaluate nerve functional recovery. At 30 days after thermal injury, the gross, microscopic, and IF staining changes of the gastrocnemius muscles were recorded in each group (Figure 5). Gross observation revealed that the gastrocnemius muscles of injured limbs were atrophic in the moderate and severe groups. Additionally, the weight of gastrocnemius muscles were 2.35 ± 0.09 g, 1.69 ± 0.08 g, and 1.26 ± 0.05 g in the slight, moderate, and severe thermal injury groups, respectively, with significant differences found. Consistently, the H&E staining results indicated that the degree of atrophy in skeletal muscles in each group was ranked as follows: slight group < moderate group < severe group. Under the light microscope, skeletal muscle cells in the severe group presented with loss of the cytoplasm, and the myofibers were sparse and separated by large distances, with increased fibrosis and fatty infiltration.

The number of intact NMJ is used as a vital index to evaluate functional nerve recovery. As an essential constituent of NMJ, neurofilaments in the gastrocnemius muscle were evaluated via IF staining to estimate the number of NMJ. The number of neurofilaments varied and was ranked in the following order: slight group > moderate group > severe group, with significant differences. Quantitatively, there was no significant difference in the number of NMJ between the slight thermal injury group and the control. However, in the moderate thermal injury group, 58 ± 2% of NMJ was fluorescently present compared to the control group. Additionally, there was only 9 ± 8% NMJ present in the severe group compared to the control group.

### 3.4. Neural Fiber Changes at 30 Days after Thermal Injury

**Figure 6:**
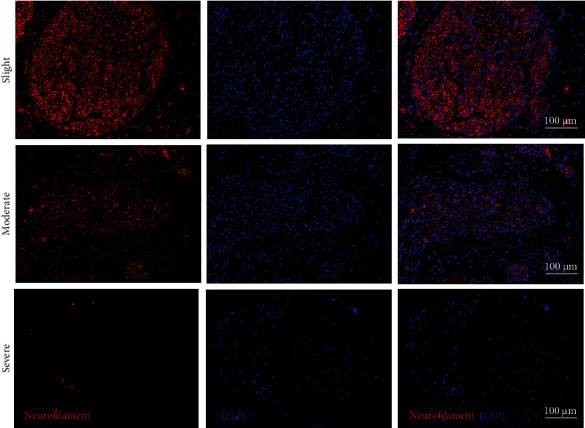
Number of neural fiber changes by IF staining (indexes: neurofilament and DAPI), at 30 days after slight, moderate, and severe thermal injury. Nuclei were stained blue using DAPI, and neural fibers stained red using antineurofilament. IF: immunofluorescent.

To evaluate the functional recovery of the sciatic nerve, the number of neural fibers was recorded by IF staining (antineurofilament) at 30 days after thermal injury (Figure 6). IF evidence revealed that the fluorescence intensity was significantly decreased afterwards in the moderate and severe thermal injury groups, while there was no significant change in the slight thermal injury group. Quantitatively, the moderate group displayed 61 ± 3% of the fluorescent intensity of the control group. And the severe group exhibited 11 ± 5% of the fluorescent intensity compared with the control group. A decrease in fluorescent intensity is assumed to correspond with the decrease in axon count, suggesting that the number of neural fibers was decreased significantly in the moderate and severe thermal injury groups (Table 2).

### 3.5. Peripheral Nerve Injury Environment after Thermal Injury

**Figure 7:**
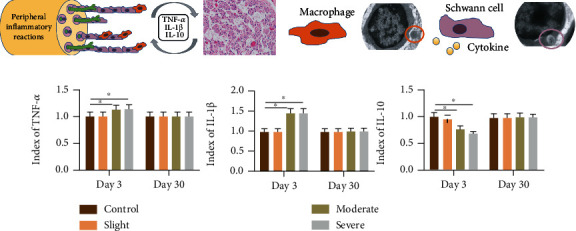
This consists of peripheral nerve injury environment. (a) Peripheral inflammatory reactions were observed at three days in the moderate and severe groups. (b) Macrophages were activated, showing tendency to be near Schwann cells in the moderate group. (c) Level of inflammatory cytokines varied in the control, slight, moderate, and severe groups. Red ellipse indicates the nucleus of the macrophage. Purple ellipse indicates the Schwann cell. ∗ represents *P* < 0.05.

The peripheral nerve injury environment consists of axons, Schwann cells, macrophages, and components of the extracellular matrix (ECM), such as neurotrophic growth factors (secreted by Schwann cells) and inflammatory cytokines (secreted by macrophages) (Figure 7(a)). Neural inflammation was obtained by H&E staining after moderate and severe thermal injury, but there was no visible reaction in the slight group. The TEM results demonstrated that macrophages were activated and showed a tendency of Schwann cells to be activated, participating in inflammatory reactions in 3 days after thermal injury (Figure 7(b)). The number of Schwann cells and macrophages per area (100 *μ*m^2^) was measured in the scope of TEM, and the results revealed that the number of macrophages was increased significantly, showing the tendency of Schwann cells to activate at 3 days after thermal injury in the moderate group. Moreover, as exhibited in Figure 7(c), the level of serum inflammatory cytokines (secreted by macrophages) varies in the three groups. Compared with the control group (performed with a sham operation), levels of TNF-*α* and IL-1*β* were increased, while the levels of IL-10 were reduced in the moderate and severe groups at 3 days after thermal injury. However, there was no significant difference in the levels of TNF-*α*, IL-1*β*, and IL-10 in the four groups at 30 days after thermal injury.

## 4. Discussion

Spontaneous reinnervation of muscles after peripheral nerve injury has been reported in the previous studies [9, 14]. Most of these studies focused on the injury of transection, traction, and compression. However, the evidence of reinnervation after thermal injury and the underlying mechanisms are still unclear. The temperature threshold of the peripheral nerve is reportedly 58°C in rats. When the exposed temperature became higher than this threshold, the endoneurium would be destroyed, causing permanent functional damage [7]. In the present study, we found that the nerve conduction ability can be recovered spontaneously if the thermal injury degree is slight and moderate and that this process is likely regulated by inflammatory reactions, with IL-1*β* and TNF-*α* increasing and IL-10 decreasing in the peripheral nerve environment. Moreover, the severity of thermal injury influenced the degree of recovery and determined whether the nerve reinnervated spontaneously. This finding is consistent with the previous study and may be relevant to the phenomenon of nerve thermal tolerance [16].

Acquiring the acute, subacute, and chronic evidence of functional and morphological changes after thermal injury is fundamental to elucidate the process of spontaneous reinnervation. During the acute stage of thermal injury, the conductive ability of the nerve is significantly impaired, but the degree of damage varies depending on the severity of thermal injury. During the subacute stage, the conductive ability is recovered in the slight group, but not in the moderate or severe group. Pathologic results also exhibit different consequences. In the slight group, the myelin sheaths are thick, and the neural axons are maintained as the concentric structure, whereas in the moderate group, the nerves maintained the complete structural integrity of the axons and myelin sheaths, but the damaged features were exhibited as loose myelin sheaths and reduced anchoring particles. In the severe group, the nerve exhibited features of coagulation and homogenization. Moreover, multiple bubbles were observed in the neural fibers and myelin sheaths, destroying the intact structures completely. In the chronic stage, nerve conduction experienced a gradual recovery period, and NCV returned to 45% compared to the normal at 30 days after thermal injury in the moderate group. Conversely, the conductive ability of the nerve could not be recovered in the severe group. These differences are also exhibited in morphological results. The neural fluorescent intensity in the moderate group was restored to 61% compared with the control, but only 11% had been detected in the severe group, which speculated that the number of neural fibers cannot restore if nerves are exposed to severe thermal injury.

The consequence of neural transient or permanent damage varies. Therefore, it is important to find critical indices for predicting the consequence of neural reinnervation. EMG results, including NCV and signal amplitude, reflect the conductive ability of the nerve [17]. In the present study, the EMG was detected during the entire process of reinnervation, and these results were closely associated with the structures of axons and myelin sheaths. Myelin sheaths, an essential component for signal transmission, have a structure that is also a critical index to predict reinnervation [18, 19]. Although the damaged features of loose myelin sheaths were detected in the moderate group, the nerves still kept the complete structures of axons and myelin sheaths, which predicted the capability of reinnervation. In the severe group, the concentric structures of myelin sheaths were completely destroyed by multiple bubbles (generated by the evaporation of interstitial fluid in the cytoplasm), which predicted that the serious damage had broken the tolerant threshold and that nerves cannot spontaneously reinnervate in the following observation [16].

The morphological change of the gastrocnemius muscle is also recognized as a vital index in evaluating functional recovery of nerves. When nerves are exposed to thermal injury, the nerve conductive ability is destroyed. Additionally, the number of intact NMJ in skeleton muscles is also significantly decreased [14, 20]. Without the domination of nerves, the muscular fibers consequently become atrophic, which is accompanied by increased fibrosis and fatty infiltration [21]. Therefore, the degree of nerve recovery can be estimated by assessing morphological changes and the number of NMJ in the skeletal muscles. As presented in this study, the number of intact NMJ increased to 58% in the moderate group compared to the control at 30 days after thermal injury, while only 9% NMJ was found in the severe group. Furthermore, the weight of the gastrocnemius muscle (representing atrophy degree) was ranked in the following order: slight group > moderate group > severe group. All of these observations are consistent with the results of EMG and nerve morphological changes discussed above.

Although it has been demonstrated that the nerve itself contributes to spontaneous reinnervation in the moderate group, neural function cannot recover at an early stage. Conversely, it is observed that nerve conduction ability was continuously damaged in 3 days after thermal injury. This abnormal finding is supported by a previous study, which can be explained by the theory of local inflammation after peripheral nerve injury [7, 22, 23]. Moreover, the existence of inflammatory events after peripheral nerve injury was also confirmed [24–26]. Additionally, it was found that the anti-inflammatory effect of steroids would inhibit lipid peroxidation, which alleviated neural dysfunction after peripheral nerve injury [27]. These studies focus on the heat conduction injury. However, the neural inflammatory mechanisms resulting from exposure to thermal injury remain unclear. Our study is primarily aimed at elucidating the underlying peripheral inflammatory reactions after thermal injury. At 3 days after thermal injury, macrophages belonging to the type of M1 at the acute stage of thermal injury in the peripheral nerve environment were observed to be proliferating and exhibiting the tendency to be near myelin sheaths. These results are consistent with the recruitment of macrophages in peripheral inflammatory reactions after neural injury [24]. Furthermore, the inflammatory reaction was also confirmed by the results of serum cytokine release, such as the high expression of IL-1*β* and TNF-*α* but low expression of IL-10. All of these observations indicate the existence of peripheral inflammatory reactions after thermal injury, which may interfere in the early stage of neural recovery.

This study has some limitations. Firstly, animal models of nerve thermal injury were derived from HS, which mimic the surgical environment. Though estimated using an infrared thermodetector, the actual temperature cannot be measured to evaluate the severity of thermal injury. Secondly, the mechanisms of inflammatory reactions, especially the processes of macrophagic activation and proinflammatory cytokine release after thermal injury, warrant further investigation.

However, despite these limitations, our study first investigates the process of neural spontaneous reinnervation after thermal injury by trying to elucidate underlying peripheral inflammatory reactions. Understanding the effects and mechanisms of spontaneous peripheral nerve reinnervation after thermal injury is helpful in bridging the subjects of medicine, biology, and bioengineering to improve reinnervation. In recent decades, various promising efforts in biology and bioengineering have been explored to improve reinnervation, including nerve conduits using an extracellular matrix, gene therapy with growth factors, and even 3D printed scaffolds [28–30]. However, these efforts are always nondirectional, without a clear path. It is critical that we determine how to promote the beneficial factors for reinnervation while preventing the harmful ones [31, 32]. Therefore, our effort to obtain a comprehensive understanding of the spontaneous reinnervation after thermal injury would be conducive to elucidating the underlying mechanism, allowing physicians to select appropriate treatments in the future.

## 5. Conclusion

Our results showed that the consequence of spontaneous peripheral nerve reinnervation varies, depending on the severity of thermal injury. When exposed to slight injury, neural function would recover in the acute stage. When exposed to moderate injury, nerve conductive ability is damaged significantly, requiring a gradual recovery period in the subacute and chronic stages. Lastly, when exposed to severe injury, both neural structures and functions were completely destroyed and were unable to reinnervate at all. Moreover, thermal injury generated inflammatory reactions in the peripheral nerve, which may interfere with neural recovery at the acute stage.

## Figures and Tables

**Table 1:** Comparison of acute nerve changes after slight, moderate, and severe thermal injury (3 days).

	Slight (*n* = 8)	Moderate (*n* = 8)	Severe (*n* = 8)	*P* value
EMG				
NCV index	0.85 ± 0.04	0.23 ± 0.04	0.06 ± 0.07	<0.01
Amplitude decrease > 50%	0/8 (0.0%)	6/8 (75.0%)	8/8 (100%)	<0.01
Latency prolongation > 10%	0/8 (0.0%)	5/8 (62.5%)	8/8 (100%)	<0.01
Neural morphology changes				
Myelin sheath: crush	0/8 (0.0%)	6/8 (75.0%)	8/8 (100%)	<0.01
Myelin sheath: bubbles	0/8 (0.0%)	5/8 (62.5%)	8/8 (100%)	<0.01
Anchoring particles: lessen	0/8 (0.0%)	4/8 (50.0%)	8/8 (100%)	<0.01
Neural inflammation				
Myelin sheath effusion	0/8 (0.0%)	5/8 (62.5%)	8/8 (100%)	<0.01
Macrophage activation	0/8 (0.0%)	6/8 (75.0%)	8/8 (100%)	<0.01
Neural homogenization	0/8 (0.0%)	4/8 (50.0%)	8/8 (100%)	<0.01

**Table 2:** Comparison of chronic changes after slight, moderate, and severe thermal injury (30 days).

	Slight (*n* = 8)	Moderate (*n* = 8)	Severe (*n* = 8)	*P* value
EMG				
NCV index	0.97 ± 0.05	0.45 ± 0.04	0.06 ± 0.08	<0.01
Amplitude decrease > 50%	0/8 (0.0%)	1/8 (12.5%)	8/8 (100%)	<0.01
Latency prolongation > 10%	0/8 (0.0%)	1/8 (12.5%)	8/8 (100%)	<0.01
Neural morphology changes				
Myelin sheath: crush	0/8 (0.0%)	1/8 (12.5%)	8/8 (100%)	<0.01
Myelin sheath: bubbles	0/8 (0.0%)	0/8 (0.0%)	0/8 (0.0%)	1.00
Anchoring particles: lessen	0/8 (0.0%)	1/8 (12.5%)	8/8 (100%)	<0.01
Inflammatory reactions	0/8 (0.0%)	0/8 (0.0%)	0/8 (0.0%)	1.00
Muscular morphology changes				
Gross weight	2.35 ± 0.09	1.69 ± 0.08	1.26 ± 0.05	<0.01
Muscular atrophy	0/8 (0.0%)	7/8 (87.5%)	8/8 (100%)	<0.01
NMJ index	0.98 ± 0.04	0.58 ± 0.02	0.09 ± 0.08	<0.01
Neural axons	0.98 ± 0.04	0.61 ± 0.03	0.11 ± 0.05	<0.01

## Data Availability

Datasets analyzed during the current study are available from the corresponding author on reasonable request.

## Abbreviations

EBDs: Energy-based devices
EMG: Electromyography
NMJ: Neuromuscular junctions
SD: Sprague-Dawley
NCV: Nerve conduction velocity
H&E: Hematoxylin and eosin
IF: Immunofluorescent
TEM: Transmission electron microscopy
ELISA: Enzyme-linked immunosorbent assay
DAPI: 4,6-Diamidino-2-phenylindole
ANOVA: One-way analysis of variance
ECM: Extracellular matrix.

## References

[1] Chiang F. Y., Lee K. D., Tae K. (2019). Comparison of hypocalcemia rates between ligasure and clamp-and-tie hemostatic technique in total thyroidectomies. *Head & Neck*.

[2] Yang X., Cao J., Yan Y. (2017). Comparison of the safety of electrotome, harmonic scalpel, and ligasure for management of thyroid surgery. *Head & Neck*.

[3] Pogorelić Z., Katić J., Mrklić I. (2017). Lateral thermal damage of mesoappendix and appendiceal base during laparoscopic appendectomy in children: comparison of the harmonic scalpel (ultracision), bipolar coagulation (ligasure), and thermal fusion technology (miseal). *The Journal of Surgical Research*.

[4] Cintron-Colon A. F., Almeida-Alves G., VanGyseghem J. M., Spitsbergen J. M. (2022). GDNF to the rescue: GDNF delivery effects on motor neurons and nerves, and muscle re-innervation after peripheral nerve injuries. *Neural Regeneration Research*.

[5] Lynch C. D., Pollock M. (1998). Chapter 21 nerve thermal injury. *Progress in Brain Research*.

[6] Tamai K., Suzuki A., Takahashi S. (2017). The incidence of nerve root injury by high-speed drill can be reduced by chilled saline irrigation in a rabbit model. *Bone & Joint Journal*.

[7] Xu D., Pollock M. (1994). Experimental nerve thermal injury. *Brain*.

[8] Lin Y. C., Dionigi G., Randolph G. W. (2015). Electrophysiologic monitoring correlates of recurrent laryngeal nerve heat thermal injury in a porcine model. *Laryngoscope*.

[9] Brookes S., Voytik-Harbin S., Zhang H., Zhang L., Halum S. (2019). Motor endplate-expressing cartilage-muscle implants for reconstruction of a denervated hemilarynx. *Laryngoscope*.

[10] Bergmeister K. D., Aman M., Muceli S. (2019). Peripheral nerve transfers change target muscle structure and function. *Science Advances*.

[11] Mu L., Sobotka S., Chen J., Nyirenda T. (2018). Nerve growth factor and basic fibroblast growth factor promote reinnervation by nerve-muscle-endplate grafting. *Muscle & Nerve*.

[12] Dubovy P., Jancalek R., Kubek T. (2013). Role of inflammation and cytokines in peripheral nerve regeneration. *International Review of Neurobiology*.

[13] Ehmedah A., Nedeljkovic P., Dacic S. (2019). Vitamin b complex treatment attenuates local inflammation after peripheral nerve injury. *Molecules*.

[14] Rosko A. J., Kupfer R. A., Oh S. S., Haring C. T., Feldman E. L., Hogikyan N. D. (2018). Immunohistologic analysis of spontaneous recurrent laryngeal nerve reinnervation in a rat model. *Laryngoscope*.

[15] Hsu M. N., Liao H. T., Truong V. A. (2019). Crispr-based activation of endogenous neurotrophic genes in adipose stem cell sheets to stimulate peripheral nerve regeneration. *Theranostics*.

[16] Carlander J., Johansson K., Lindström S., Velin A. K., Jiang C. H., Nordborg C. (2005). Comparison of experimental nerve injury caused by ultrasonically activated scalpel and electrosurgery. *The British Journal of Surgery*.

[17] Shrager P. (1988). Ionic channels and signal conduction in single remyelinating frog nerve fibres. *The Journal of Physiology*.

[18] Geren B. B., Schmitt F. O. (1954). The structure of the Schwann cell and its relation to the axon in certain invertebrate nerve fibers. *Proceedings of the National Academy of Sciences of the United States of America*.

[19] Liu B., Xin W., Tan J. R. (2019). Myelin sheath structure and regeneration in peripheral nerve injury repair. *Proceedings of the National Academy of Sciences of the United States of America*.

[20] Kupfer R. A., Old M. O., Oh S. S., Feldman E. L., Hogikyan N. D. (2013). Spontaneous laryngeal reinnervation following chronic recurrent laryngeal nerve injury. *Laryngoscope*.

[21] Tews D. S. (2005). Muscle-fiber apoptosis in neuromuscular diseases. *Muscle & Nerve*.

[22] Mohammadi R., Azad-Tirgan M., Amini K. (2013). Dexamethasone topically accelerates peripheral nerve repair and target organ reinnervation: a transected sciatic nerve model in rat. *Injury*.

[23] Siqueira Mietto B., Kroner A., Girolami E. I., Santos-Nogueira E., Zhang J., David S. (2015). Role of IL-10 in resolution of inflammation and functional recovery after peripheral nerve injury. *The Journal of Neuroscience*.

[24] Gaudet A. D., Popovich P. G., Ramer M. S. (2011). Wallerian degeneration: gaining perspective on inflammatory events after peripheral nerve injury. *Journal of Neuroinflammation*.

[25] Zhang D., Sun H., Tufano R., Caruso E., Dionigi G., Kim H. Y. (2020). Recurrent laryngeal nerve management in transoral endoscopic thyroidectomy. *Oral Oncology*.

[26] Zhang R. R., Chen S. L., Cheng Z. C., Shen Y. Y., Yi S., Xu H. (2020). Characteristics of cytokines in the sciatic nerve stumps and DRGs after rat sciatic nerve crush injury. *Military Medical Research*.

[27] Galloway E. R., Jensen R. L., Dailey A. T., Thompson B. G., Shelton C. (2000). Role of topical steroids in reducing dysfunction after nerve injury. *Laryngoscope*.

[28] Chen C. C., Yu J., Ng H. Y. (2018). The physicochemical properties of decellularized extracellular matrix-coated 3d printed poly(epsilon-caprolactone) nerve conduits for promoting schwann cells proliferation and differentiation. *Materials*.

[29] Dixon A. R., Jariwala S. H., Bilis Z., Loverde J. R., Pasquina P. F., Alvarez L. M. (2018). Bridging the gap in peripheral nerve repair with 3d printed and bioprinted conduits. *Biomaterials*.

[30] Liao C. F., Chen C. C., Lu Y. W. (2019). Effects of endogenous inflammation signals elicited by nerve growth factor, interferon-*γ*, and interleukin-4 on peripheral nerve regeneration. *Journal of Biological Engineering*.

[31] Hernandez-Morato I., Tewari I., Sharma S., Pitman M. J. (2016). Blockade of glial-derived neurotrophic factor in laryngeal muscles promotes appropriate reinnervation. *Laryngoscope*.

[32] Nagappan P. G., Chen H., Wang D. Y. (2020). Neuroregeneration and plasticity: a review of the physiological mechanisms for achieving functional recovery postinjury. *Military Medical Research*.

